# Fabrication and biological investigation of a novel star polymer based on magnetic cyclic aromatic polyimide chains

**DOI:** 10.1038/s41598-023-36619-x

**Published:** 2023-06-13

**Authors:** Reza Eivazzadeh-Keihan, Zahra Sadat, Adibeh Mohammadi, Hooman Aghamirza Moghim Aliabadi, Amir Kashtiaray, Ali Maleki, Mohammad Mahdavi

**Affiliations:** 1 Catalysts and Organic Synthesis Research Laboratory, Department of Chemistry, Iran University of Science and Technology, Tehran, 16846-13114 Iran; 2 Advanced Chemical Studies Lab, Department of Chemistry, K. N. Toosi University of Technology, Tehran, Iran; 3 Endocrinology and Metabolism Research Center, Endocrinology and Metabolism Clinical Sciences Institute, Tehran University of Medical Sciences, Tehran, Iran

**Keywords:** Biotechnology, Chemistry, Materials science, Nanoscience and technology

## Abstract

Herein, a novel nanostructure based on cyclic aromatic polyimide with statistical star polymer structure was synthesized via the functionalization of the CuFe_2_O_4_ MNPs surface. The polymerization process on the functionalized surface of CuFe_2_O_4_ MNPs was performed with pyromellitic dianhydride and phenylenediamine derivatives. All analytical methods such as Fourier-transform infrared (FT-IR) spectroscopy, thermogravimetric (TG) analysis, X-ray diffraction (XRD) pattern, energy-dispersive X-ray (EDX), field-emission scanning electron microscope (FE-SEM), vibrating-sample magnetometer (VSM) were performed to characterize the structure of CuFe_2_O_4_@SiO_2_-polymer nanomagnetic. The cytotoxicity of CuFe_2_O_4_@SiO_2_-Polymer was investigated for biomedical application by MTT test. The results proved that this nanocmposite was biocompatible with HEK293T healthy cells. Also, the evaluation antibacterial property of CuFe_2_O_4_@SiO_2_-Polymer showed that its MIC in Gram-negative and Gram-positive bacteria were 500–1000 µg/mL, so it had antibacterial activity.

## Introduction

Recent advances in controlled polymerization have made a wide range of complex architectures with specific molecular weights for a variety of applications. Star polymers have been widely studied due to their unique structure and application in advanced materials^1^. Star polymers are known as a class of branched macromolecules. They consist of a central nucleus and linear polymer branches fused to the center point. These polymers are classified into two categories, homogeneous and heterogeneous, based on the structure and chain length.

For further explanation, when the arms are the same in structure and length, they fall into the category of homogeneous polymers, and vice versa, when they are different in structure and length chains known as heterogeneous polymers. This class of polymers is mighty to self-assemble into supramolecular structures with extra features that can be sketched using their functionalize arms, and this has led to research interest in them^2^. These unique features, which are not available to other linear counterparts, have given them many applications in various fields including materials science, medicine, and pharmacy^3^.

So far, star polymers have been widely used in biomedical applications such as targeted drug delivery, antibacterial biomaterials, tissue engineering, diagnosis, and gene delivery. The unique structure and attractive chemical and physical properties of star polymers such as encapsulability, low viscosity in dilute solutions, increased response to stimuli, internal and environmental performance have caused them to receive much attention. Research has shown that the production of composites of magnetic nanoparticles with polymer coatings creates hybrid structures that are significantly useful in cancer therapy. Magnetic ferrite nanoparticles have been one of the most widely used particles in the biomedical. These leading nanoparticles have become one of the most important materials in various fields such as catalysis, biomedicine and nanotechnology due to their unique size-dependent properties^4^.

Nanotechnology provides a promising ground for developing nanomaterials with sizes between 1 and 100 nm and unique physicochemical properties^5^. XFe_2_O_4_ (where X = Ni, Cu, Co, Zn, Mg, etc.) magnetic nanoparticles make an important class of magnetic materials that exhibit unique optical, electronic, and magnetic properties^6^. These nanoparticles have high permeability and good saturation magnetism and are easily magnetized and lose their magnetic properties and are also electrically insulating.

Copper ferrite (CuFe_2_O_4_) nanoparticles are one of the important ferrites that show phase transfer, change semiconductor properties and electrical switch and quadrilateral changes under different conditions^7,8^. In addition to suitable magnetic, electrical, and thermal stability, these nanoparticles have a wide range of applications in catalysts^9^, lithium-ion batteries^10^, bioprocessing^11^, color imaging^12^, and gas sensing^13^. These nanoparticles also have great potential for use in biomedical application^14,15^, for example in diagnostic imaging^16,17^, drug delivery^18,19^, hyperthermia therapy^15,20–25^, and cell labeling^26^. So far, not much information is available about the biological response of the Copper ferrite in combination with other materials, and this has made the use of these nanoparticles in biomedicine a challenge.

Recently, in order to activate the surfaces of magnetic nanoparticles^27^, increase biocompatibility^28^ and colloidal stability^29^ in environmentally friendly environments and prevent their accumulation due to magnetic forces between particles, the surface of these nanoparticles is covered with natural and synthetic polymers such as cellulose, pectin, agar, chitosan, alginate, polypyrrole, and polyvinylidene fluoride^4^. In this regard, the polymerization reaction and polymer growth have been performed on the surface of magnetic nanoparticles CuFe_2_O_4_ MNPs. Under these conditions, magnetic nanoparticles act as the core of the star-shaped polymer and form magnetic star-shaped structures.

A general strategy for presenting functional molecules and the polymerization process at the surface of magnetic nanoparticles is to use anchor molecules. In the following, the polymerization process is performed radically on the surface of the modified nanoparticles using an initiator. Initiators can react with a monomer to form an intermediate compound capable of sequentially bonding a large number of other monomers to a polymeric compound. In this work, a new nanostructure was synthesized.

The CuFe_2_O_4_ as a central core was functionalized by tetraethyl orthosilicate** (**TEOS), (3-Chloropropyl)-trimethoxysilane (CPTMS), and phenylenediamine derivatives, step by step. Finally, the polymerization reaction of pyromellitic dianhydride on the surface of functionalized CuFe_2_O_4_ MNPs was performed by different phenylenediamine derivatives. All analysis were done to characterize the structural of the novel magnetic hybrid CuFe_2_O_4_@SiO_2_-cyclic aromatic polyimide. Also, its biological properties were investigated.

The result showed that the toxicity of CuFe_2_O_4_@SiO_2_-Polymer at the highest concentration was 12.64% and this nanostructure is biocompatible with HEK293T cells. Also, minimal inhibitory concentration and minimal bactericidal concentrations of CuFe_2_O_4_@SiO_2_-Polymer in comparison two control antibiotics (Penicillin and Streptomycin) was investigation against a Gram-positive bacteria (*Staphylococcus aureus*) and two Gram-negative bacteria (*Escherichia coli* and *Pseudomonas aeruginosa*). The result proved that prepared nanostructure has acceptable antibacterial activity.

A significant breakthrough has been achieved by synthesizing star polymers with a magnetic center for the first time, a feat that has been accomplished only by this particular research group. In this study, not only focused on the magnetic center but also on the arms of these star polymers, which were made up of polyimides. The combination of these two components led to a novel structure that has never been seen before.

CuFe_2_O_4_ was used as the magnetic center of the star polymer, a unique aspect of this research. The novelty of the structure is not limited to the use of CuFe_2_O_4_ alone, but also in the fact that aromatic polyimide chains were used to decorate the magnetic center in the form of a star polymer. This pioneering development marks the first time that CuFe_2_O_4_ has been decorated with polyimide chains in the form of a star polymer.

In previous studies of star polymers, the antibacterial property was not attributed to the center. However, this research found that the use of CuFe_2_O_4_ as the center of the star polymer led to the antibacterial property. This discovery highlights the importance of the center in determining the properties of star polymers.

Despite the promising benefits of using CuFe_2_O_4_ as the magnetic center, it is important to note that it could also be toxic. However, this issue was overcome by decorating it with polyimide arms, which acted as a protective layer.

Overall, this new structure of star polymers with a magnetic center decorated with polyimide arms is a significant innovation in the field. It opens up new avenues of research and has the potential for practical applications in various fields, including biomedicine and environmental science.

## Experimental

### General

All chemicals used as solvents or reagents with high purities were provided by international Sigma Aldrich and Merck companies. Various analyzes were performed to investigate the structure of magnetic star polymers and confirm the synthesis and growth of the polymer on the modified surface of the central core nanoparticles. For example, the FT-IR (Fourier-transform infrared) spectra were performed by using the KBr pellets method (Perkin Elmer spectrum RX1). The XRD (X-ray diffraction) pattern was taken by using Bruker device (D8 advance model). EDX (Energy dispersive X-ray) analysis was implemented TESCAN MIRA II Xmax device. FE-SEM (field-emission scanning electron microscope) was used to evaluation the structure, morphology, and size of designed magnetic nanostructures by TESCAN MIRA III device. The TGA (thermogravimetric analysis) and VSM (vibrating-sample magnetometer) analysis was taken to evaluate its thermogravimetric and magnetic behavior by Bahr-STA 504 under the argon atmosphere and the rate of 10 °C/min and LBKFB model-magnetic Kashan kavir (Iran) (5000 Oe) devices, respectively.

### Preparation sections of CuFe_2_O_4_@SiO_2_-polymer

#### Preparation of CuFe_2_O_4_ MNPs

In order to prepare CuFe_2_O_4_ MNPs^23^, Initially, 8.2 mmol of Fe (NO_3_)_2_·9H_2_O was dissolved in 75 mL of distilled water and 4.1 mmol of Cu (NO_3_)_2_·3H_2_O was added to the solution and kept under stirring condition to absolutely dissolve. After that, a 5 M solution of NaOH was prepared and added dropwise to the initial solution and kept under stirring condition at 90 °C for 2 h. Next, the solution was chilling to room temperature and the prepared magnetic precipitate was mustered with an external magnet and washed with distilled water for several times. Then, the fabricated magnetic nanoparticles were dried for 24 h at 80 °C. Finally, the dried magnetic nanoparticles were calcinated at 700 °C for 5 h with the heating rate of 20 °C/min.

#### Surface functionalization of CuFe_2_O_4_ MNPs using silica shell (CuFe_2_O_4_@SiO_2_)

The preparation of nanostructures CuFe_2_O_4_@SiO_2_ was performed according to the Sto¨ber method as follows: Initially, 0.22 g of magnetic nanoparticles were dispersed by ultrasonic waves in 50 mL of deionized distilled water. Afterward, 7.5 mL of 25% ammonia solution was added drop by drop to the solution under the stirring condition at room temperature. Then, 80 mL ethanol was added to the mixture solution. After ethanol was added, 4 mL of TEOS was added drop by drop to the mixture solution and kept under stirring condition at room temperature for 24 h. Finally, the prepared solid was mustered by an external magnet and washed with deionized water and ethanol, and dried for 24 h in the oven at 70 °C^23^.

#### Preparation of SiO_2_-layered CuFe_2_O_4_ MNPs coated by CPTMS layer (CuFe_2_O_4_/SiO_2_–Cl)

The activated surface of CuFe_2_O_4_ MNPs was covered by the second layer CPTMS as follows: Firstly, 0.69 g of SiO_2_/CuFe_2_O_4_ powder was added to 100 mL of dry toluene and kept under stirring at 60 °C. After a few minutes, 1 mL of CPTMS was added dropwise to the mixture solution and kept under stirring at 60 °C for 18 h. Finally, the obtained magnetic fouling was separated and dried for 12 h in the oven at 60 °C^23^.

#### Preparation of SiO_2_-layered CuFe_2_O_4_ MNPs functionalized by phenylenediamine derivatives layer (CuFe_2_O_4_ @ SiO_2_-phenylenediamine)

The functionalization of the SiO_2_-layered CuFe_2_O_4_ MNPs surface by phenylenediamine derivatives was done as follows: Firstly, 2 mmol of phenylenediamine derivative was dissolved in 25 mL of ethanol and 1.00 g of CuFe_2_O_4_@SiO_2_-Cl MNPs was poured to the mixture solution. Afterward, the mixture solution was refluxed (80 °C) for 12 h. Then, the prepared magnetic solid was separated with external magnet and washed with ethanol. Finally, the phenylenediamine-functionalized CuFe_2_O_4_ was dried in an oven at 80 °C for 12 h^23^.

#### Prepared CuFe_2_O_4_@SiO_2_-cyclic aromatic polyimide with the polymerization on the surface of modified CuFe_2_O_4_ MNPs

In this step, 1.00 gr of CuFe_2_O_4_ @ SiO_2_-phenylenediamine was dissolved at 50 mL DMF. After that, 10 mmol of phenylenediamine derivative was added to the solution and kept under stirring condition for 20 min. Next, 10 mmol of pyromellitic dianhydride was instilled to the solution during 1 h. Then, the mixture solution was kept stirred mechanically for 3 h at room temperature. Afterwards, the mixture solution was kept under refluxing conditions at the N_2_ atmosphere for 2 min. In final step, the functionalized CuFe_2_O_4_ MNPs with polyamidic acid were washed with DMF and ethanol for several times and then dried under the vacuum oven condition at 160 °C for 12 h to form polyimide.

#### MTT assay

MTT assay was used to measure the toxicity and biocompatibility of the synthesized CuFe_2_O_4_@SiO_2_-Polymer. First, HEK293T cells (human embryonic kidney cell line) were prepared from the Pasteur Institute of Iran and cultured at 1 × 10^5^ cell/well in 96 well plate under optimal conditions (37 °C, 5% CO_2_ in humidified incubator). Next, the growth media (10% FBS) was removed and the cells were washed two times with phosphate buffer saline (PBS). New maintenance Roswell Park Memorial Institute Medium (RPMI) medium (10% FBS) containing 0.5, 5, 50, 500, and 1000 µg/mL of synthesized nanostructure was added and the cells were incubated for 24, 48, and 72 h. Quintet wells were analyzed for each concentration and column elution buffer was used as the control. A 10 μL solution of freshly prepared 5 mg/mL MTT in PBS was added to each well and allowed to incubate for an additional 4 h. The media was removed and isopropanol was added at 100 µL/well. Plates were shaken gently to facilitate formazan crystal solubilization. The absorbance was measured at 545 nm using a microplate reader (STAT FAX 2100, BioTek, Winooski, USA). The percentage of toxicity and cell viability was calculated according to these formulas^14,30^.

$$Toxicity \%=\left(1- \frac{mean\,\, OD\,\, of \,\,sample}{mean \,\,OD\,\, of\,\, negative\,\, control}\right)\times 100$$

$$Viability \%=100-Toxicity \%$$

#### Antimicrobial properties

The antimicrobial activity of the CuFe_2_O_4_@SiO_2_-Polymer was evaluated using a serial dilution titration method, according to Clinical and Laboratory Standards Institute (CLSI) guidelines, to measure Minimal Inhibitory Concentration (MIC) of the synthesized nanostructure against different bacterial strains^31^. For this purpose, bacteria were grown overnight at 37 °C in Mueller Hinton Broth (MHB) and Roswell Park Memorial Institute (RPMI) 1640 medium, respectively, then were diluted in the same medium. Serial dilutions of CuFe_2_O_4_@SiO_2_-Polymer were added to the microtiter plates in a volume of 100 μL, followed by the addition of 100 μL of bacteria to give a final inoculum of 5 × 10^5^ colony-forming units (CFU)/mL. The plates were incubated at 37 °C for 24 h and 48 h, and the MICs were determined. Then, 100 μL of the initial bacteria inoculums of 5 $\times$ 10^5^ CFU/mL were platted on Mueller–Hinton Agar (MHA) and Sabouraud *Maltose* Agar (SMA) as the positive control, and 100 µL of the 24 h inhibitory concentration test samples was platted on same media to determine the minimal bactericidal concentrations (MBCs)^14,32^.

## Results and discussion

**Figure 1:**
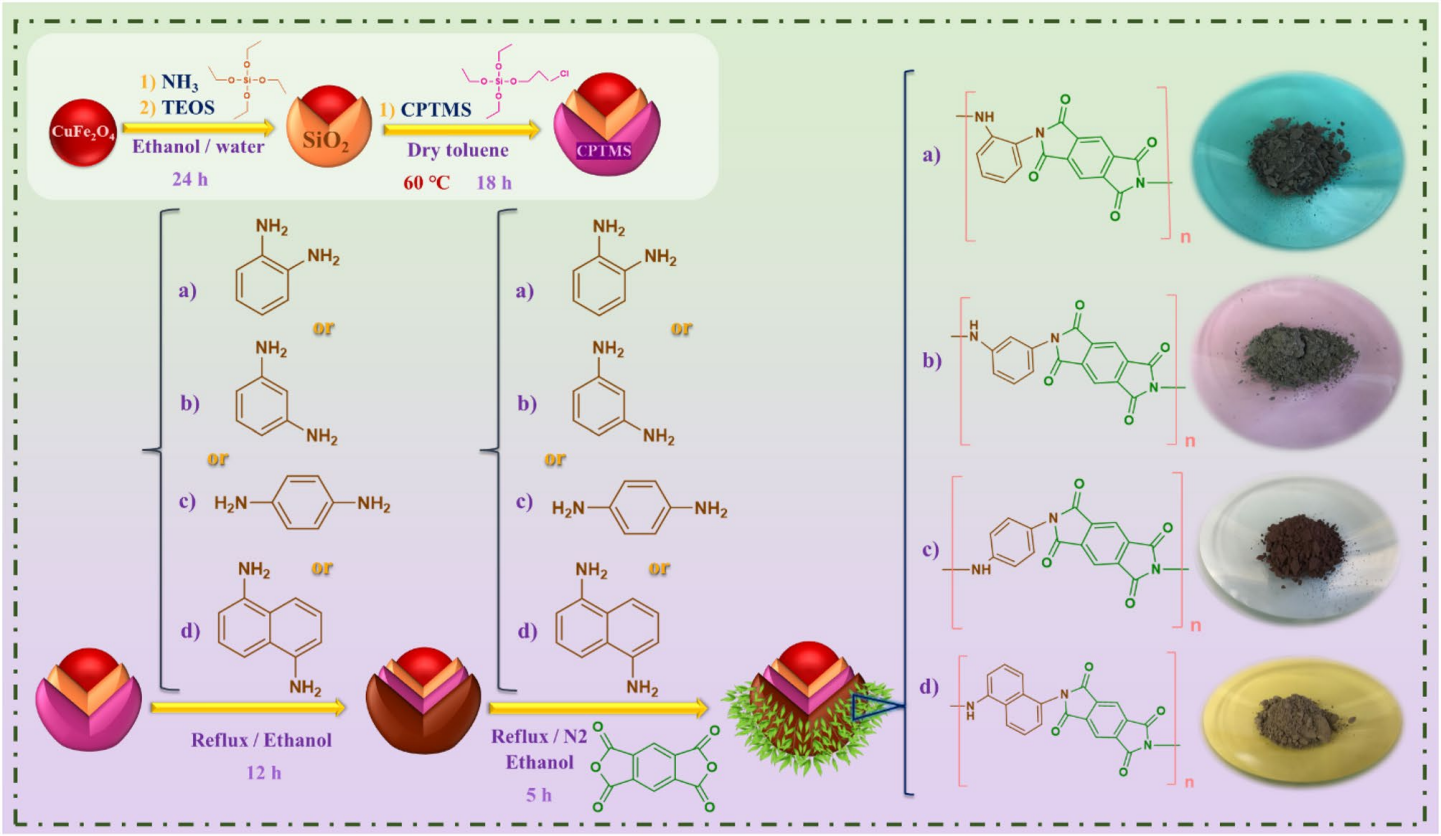
The synthesis process of CuFe_2_O_4_@SiO_2_-polymer nanostructure.

The intended nanostructure was synthesized for applying in biological application. The CuFe_2_O_4_ nanoparticle was chosen as the central core. According to reports, copper ferrite nanoparticles in combination with other metals have shown significant antibacterial properties^33^, but these nanoparticles can cause dose-dependent cytotoxicity to healthy cells^34^. Functionalizing the surface of these nanoparticles and forming a star-shaped polymer coating minimized the toxicity of these nanoparticles and formed a biocompatible nanostructure. As stated in the experimental section, the surface of these nanoparticles was functionalized by TEOS, CPTMS and phenylenediamine derivatives in three stages. Then, polyamidic acid was performed by pyromellitic dianhydride and phenylenediamine derivatives on the surface of these functionalized nanoparticles. Finally, the polyimide acid was imidized into cyclic aromatic polyimide by in-situ annealing treatment at 160 °C. All synthesis steps of CuFe_2_O_4_@SiO_2_-cyclic aromatic polyimide and the polymerization process are well shown in Fig. 1. The types of analytical and spectral analyses were performed to characterize the structural of CuFe_2_O_4_@SiO_2_-polymer nanostructure. The new functional groups was evaluated by FT-IR spectrum. The TGA analysis was studied to evaluate the thermal behavior. The elemental composition was investigated by EDX spectrum. The XRD patterns and VSM analyses were perused to specify the crystalline phase and magnetic properties of the prepared nanostructure. Finally, the cytotoxicity and antimicrobial properties of CuFe_2_O_4_@SiO_2_-polymer nanostructures were evaluated to apply for biomedical application.

### FT-IR and TGA analyses

**Figure 2:**
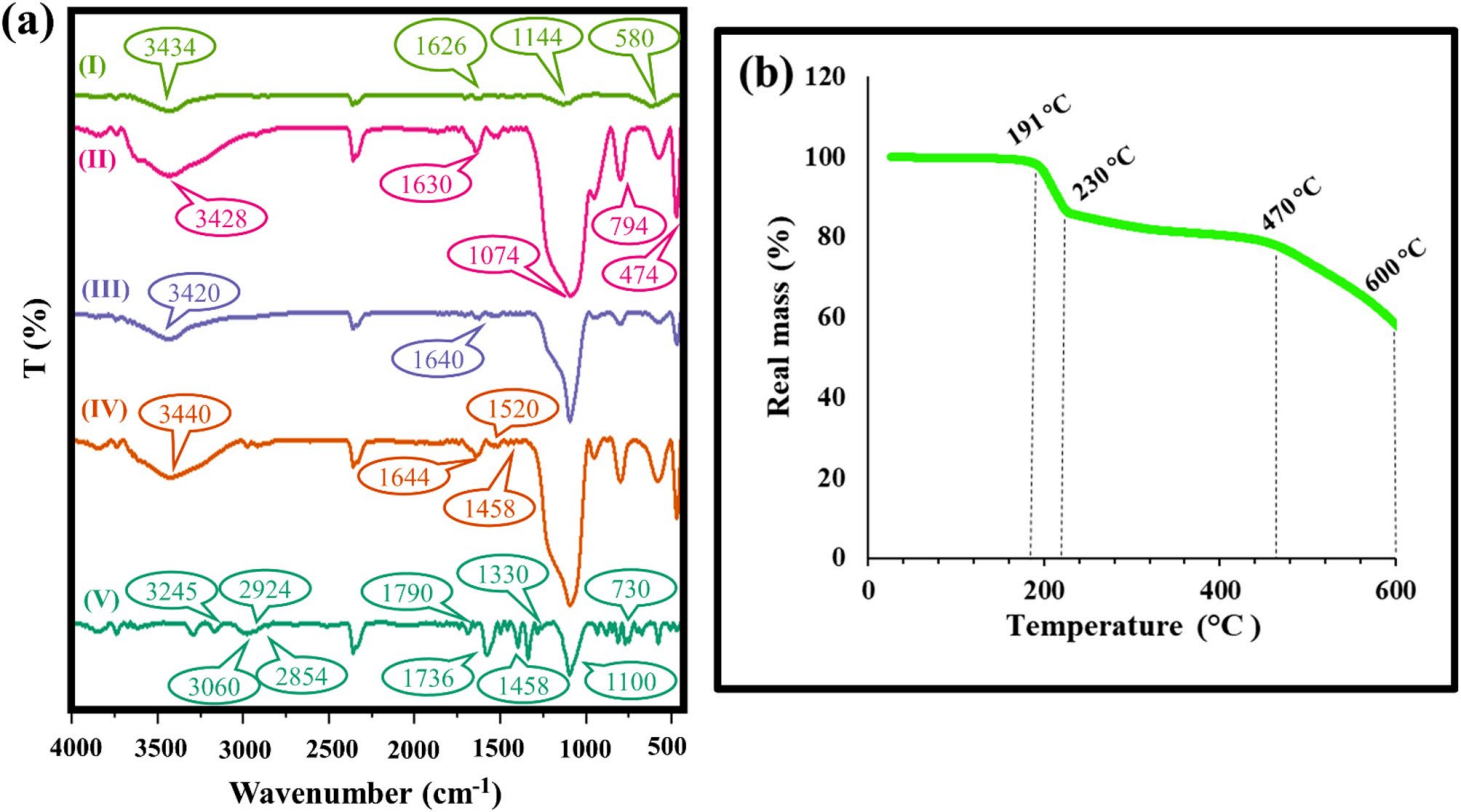
FT-IR spectra of (I) CuFe_2_O_4_ MNPs, (II) CuFe_2_O_4_@SiO_2_, (III) CuFe_2_O_4_@CPTMS, (IV) CuFe_2_O_4_/SiO_2_-(phenylenediamine), and (V) magnetic CuFe_2_O_4_@SiO_2_-polymer nanostructure (**a**), and TGA analysis of the prepared nanostructure (**b**).

The FT-IR spectrum of CuFe_2_O_4_ MNPs was showed at Fig. 2a(I). The absorbance band appeared around 580 cm^−1^ attributed to vibration modes of Fe–O and Cu–O bonds (tetrahedral complexes) in CuFe_2_O_4_ MNPs^23^. A small and broad band was assigned around 1114 cm^−1^ related to the stretching vibration of the hexagonal sites in the crystalline structure of metal oxide. Also, the broad absorbance band at 3434 cm^−1^ showed the stretching vibration of OH groups on the surface of MNPs. Figure 2a (II) displays the FT-IR spectrum of CuFe_2_O_4_@SiO_2_ which new functional groups appeared. Three absorbance bands at 491, 794, and 1074 cm^−1^ supported the presence of Si–O–Si asymmetric and symmetric stretching vibrations and also bending vibration modes. In addition, two broad bands were observed at 3420 cm^−1^ and 1630 cm^−1^ attributed to the O–H stretching vibration mode and O–H stretching vibration of Si–OH in the silica shell^4^. Figure 2(III) demonstrates the FT-IR spectrum of CuFe_2_O_4_@SiO_2_–Cl and new functional groups. The absorbance band illustrated around 1480 cm^−1^ pertaining to the Si–CH_2_ stretching vibration mode. A significant decrease was observed in all of the peaks at the CuFe_2_O_4_@SiO_2_–Cl spectrum compared to the previous step attributed to the anchor propyl groups. Figure 2a(IV) demonstrates the FT-IR spectra of CuFe_2_O_4_/SiO_2_-(phenylenediamine) as the third layer. Two broad absorbance bands appeared around 1458, and 1644 cm^−1^ attributed to the stretching vibration mode of quinonoid and benzenoid rings, respectively. Also, the N–H stretching vibration appeared as broad absorbance band around 3440 cm^−1^^21^. The FT-IR spectra of CuFe_2_O_4_@SiO_2_-polymer nanostructure was displayed at Fig. 2a(V). In the Fourier transform infrared (FTIR) spectrum of polyimides, distinctive absorption bands are observed at wavenumbers of around 1100 cm^−1^ and 730 cm^−1^. The absorption band at 1100 cm^−1^ is attributed to the out-of-plane C–N bending vibration of the (O=C)2–N group in the imide ring^35,36^, while the absorption band at 730 cm^−1^ is typically attributed to the CN imide ring deformation^37,38^. These C–N bending motions are characteristic features of the imide functional group, which is present in the backbone of polyimides. The intensity and position of these absorption bands can provide valuable information about the degree of polymerization, the extent of imidization, and the overall chemical structure of the polyimide material. Therefore, the identification and interpretation of these C–N bending peaks in the FTIR spectrum is an important tool for the analysis and characterization of polyimides^39,40^. Also, in the imidization process, the stretching vibrations of C=C double bond of benzene ring and C–N–C bond were appeared around 1458 cm^−1^ and 1330 cm^−1^, respectively^41–43^. The two peaks appearing at frequencies 2854 and 2924 cm^−1^ were as a result of the C–H symmetric and asymmetric stretching bonds in the aliphatic hydrocarbons^44^. In addition, an absorbance peak around 3060 cm^−1^ probably attributed to *sp*^2^ C–H symmetric and asymmetric stretching bonds^42^. The FT-IR spectrums of the other phenylenediamine derivatives have been shown in Fig. S1 and attached in the supplementary information. The thermal stability of CuFe_2_O_4_@SiO_2_-cyclic aromatic polyimide nanostructure was evaluated by thermogravimetric analysis in a thermal range of 50–600 °C, shown in Fig. 2b. As shown, three distinct reduction peaks is observed in the TGA diagram. The first reduction peak (12%) appeared at 191 °C to 230 °C range temperature which it can be related to the adsorbed solvent molecules and impurities in the structure of magnetic nanostructure star polymer. The second reduction peak (10%) occurred at 230 °C to about 470 °C and it is due to decomposition of organic part of the molecule and grafted linkers^45^. Finally, the third reduction peak (20%) was seen at 470 °C to 600 °C range temperature and it is assigned to the decomposition of fabricated polymer structure^14^. Also, in Fig. S2, the TGA analyses of the various phenylenediamine derivatives have been illustrated (Supplementary information).

### XRD and VSM analyses

**Figure 3:**
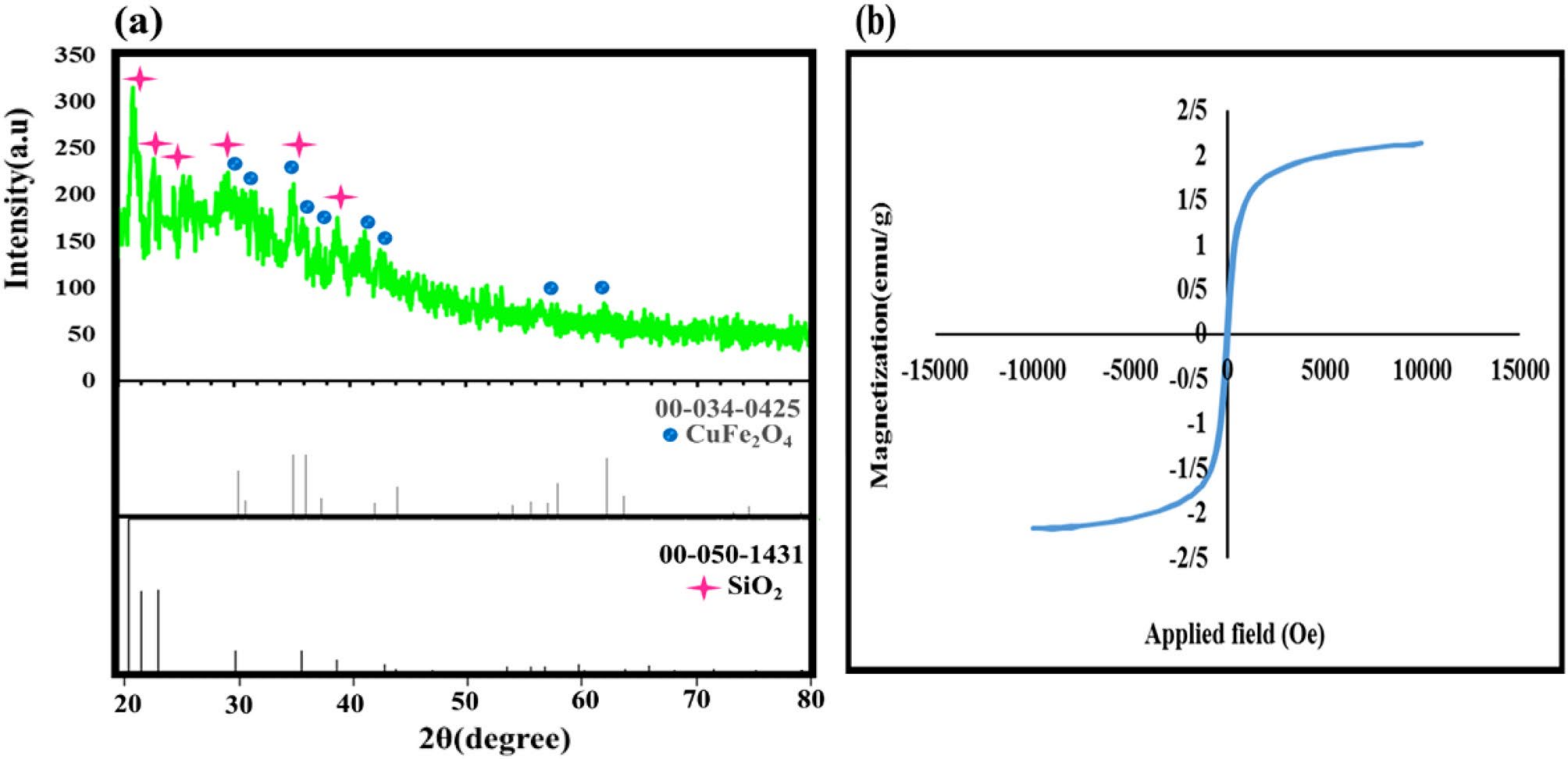
The XRD pattern (**a**) and VSM analysis of CuFe_2_O_4_@SiO_2_-polymer nanostructure (**b**).

The XRD pattern of synthesized magnetic CuFe_2_O_4_@SiO_2_-cyclic aromatic polyimide nanostructure was demonstrated in Fig. 3a. As shown, the typical diffraction peaks of (2θ = 29.50, 30.65, 34.88, 35.20, 37.23, 41.29, 43.52, 57.87, 62.13) attributed to the lattice planes (112), (200), (103), (211), (202), (004), (220), (321), (224) confirmed the presence of CuFe_2_O_4_ MNPs (JCPDS card No.00-034-0425). Also, the other diffraction signals at (2θ = 20.50, 21.85, 22.96, 29.50, 35.12, 38.90) related to the lattice planes (100), (002), (101), (102), (110), (103) which confirmed the presence of SiO_2_ shell (JCPDS card No.00-046-1045).The XRD pattern of the phenylenediamine derivatives has been displayed in the Fig. S3 (supporting information). The characteristic magnetic of prepared nanostructure was investigated by VSM analysis by applying a magnetic field between − 15 < k Oe <  + 15 showing in Fig. 3b. Several parameters including the crystalline structure of magnetic nanoparticles, core size, shell thickness, and the distance between particles are effective in the magnetization property of core–shell structures. According previous works, the value of saturation magnetization of CuFe_2_O_4_ MNPs was between range of 20 and 30 emu g^−1^^23^. Figure 3b displays the value saturation magnetization of CuFe_2_O_4_@SiO_2_-cyclic aromatic polyimide nanostructure star polymer, which is decreased to 2.13 emu. g^-1^ due to the surface functionalization of core CuFe_2_O_4_ MNPs. In addition, the VSM analyses of the phenylenediamine derivatives has been shown in the Fig. S4 (Supporting Information).

### FE-EEM and EDX analyses

**Figure 4:**
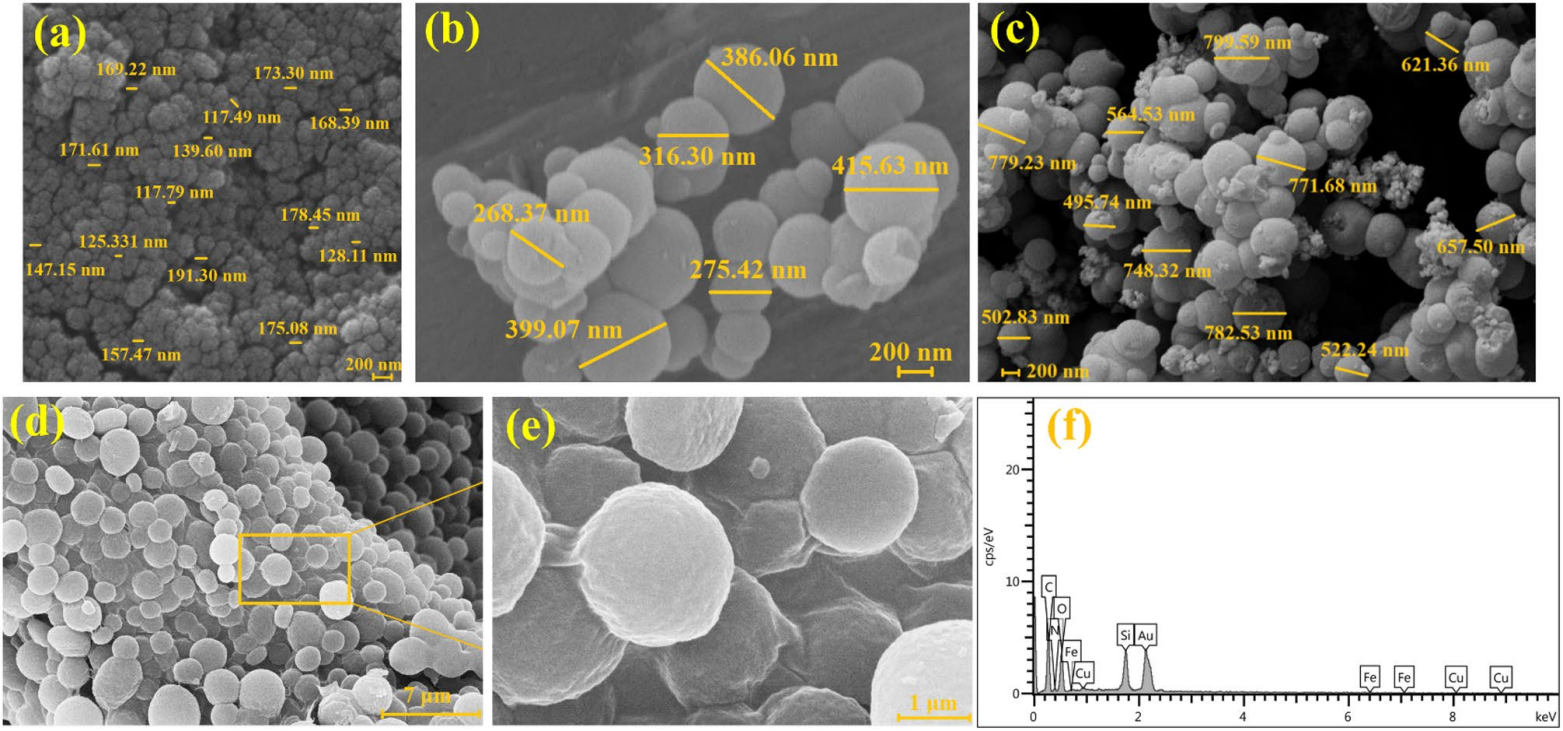
FE-SEM images of CuFe2O4 (**a**), CuFe_2_O_4_/SiO_2_–Cl (**b**), CuFe_2_O_4_ @ SiO_2_-phenylenediamine (**c**), CuFe_2_O_4_@SiO_2_-polymer nanostructure (**d**, **e**) and EDX spectrum (**f**) of CuFe_2_O_4_@SiO_2_-polymer nanostructure.

Figure 4 contains FES-EM images depicting various stages of the synthesis and resulting products. Figure 4a shows the presence of spherical nanoparticles with an average diameter of 150 nm, which belong to the pure copper ferrite nanoparticles synthesized. In the next stage, after the deposition of silica layers, the average diameter of nanoparticles increased to 300–400 nm, as shown in Fig. 4b. This stage is the main reason for the reduction in toxicity of raw copper ferrite nanoparticles in the final structure. Subsequently, after functionalizing the structure with phenylenediamine derivatives, the average diameter of nanoparticles further increased. As evident in Fig. 4c, the average diameter of the nanostructure exceeded 500 nm. Further, FE-SEM images was used to investigate the morphology and polymer growth around the CuFe_2_O_4_ central core and it is shown in Fig. 4d and e. As shown, the grown magnetic nanoparticles have a spherical morphology. The increase in the size of the spherical particles is due to the progressive growth of the cyclic aromatic polyimide chains around the magnetic cores. Additionally, it is noteworthy that the average diameter of the spherical nanostructures has exceeded 1 μm. In order to check the quality of the constituent elements of the designed nanostructure, EDX spectrum was prepared. As seen in Fig. 4f, the three Fe, Cu, and O peaks are related to the CuFe_2_O_4_ MNPs as central core. In addition, the presence of Si and N peaks is attributed to the functionalization of magnetic nanoparticles with SiO_2_ and CPTMS. The C and N peaks in the spectrum confirmed of the implementation the polymerization process of cyclic aromatic polyimide chains in the presence of phenylenediamine derivatives.

### Cytotoxicity

**Figure 5:**
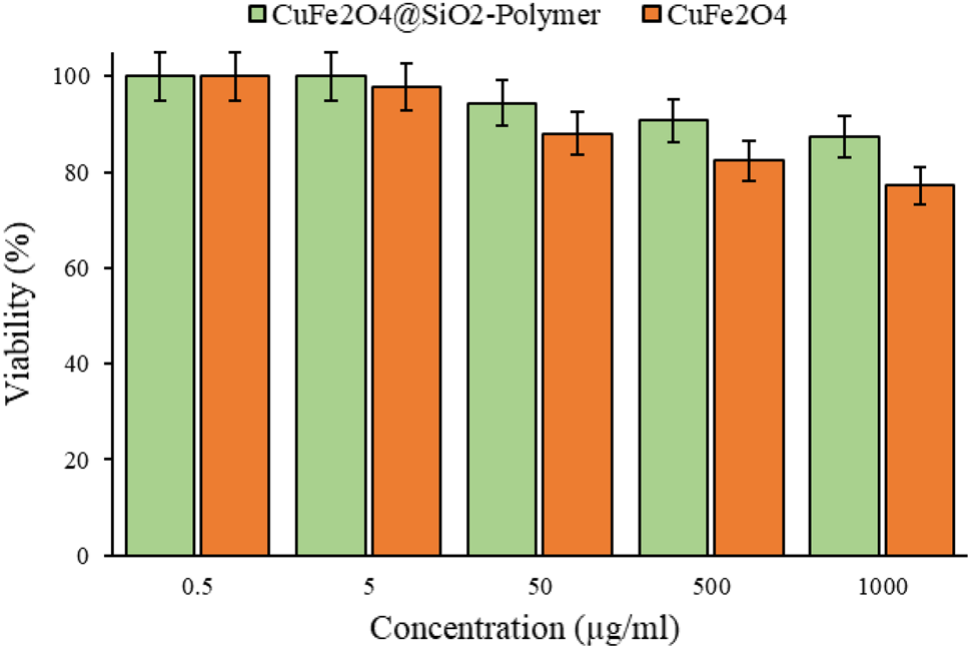
This histogram shows the HEK293T cells viability percentage at different concentrations of CuFe_2_O_4_@SiO_2_-Polymer and CuFe_2_O_4_ nanoparticles.

The toxicity and cell viability of CuFe_2_O_4_@SiO_2_-Polymer at the highest concentration (1000 μg/mL) were 12.64% and 87.36%, respectively (Fig. 5). The results are the average of three independent experiments. This indicates that CuFe_2_O_4_@SiO_2_-Polymer is biocompatible with HEK293T cells. The results showed that the toxicity of crude nanoparticles is higher than the final composite. In fact, placing these nanoparticles in the composite has reduced their toxicity. This histogram shows the HEK293T cells viability percentage at different concentrations of CuFe_2_O_4_@SiO_2_-Polymer and CuFe_2_O_4_ nanoparticles.

### MIC and MBC determination

MICs and MBCs of CuFe_2_O_4_@SiO_2_-Polymer and two control antibiotics (Penicillin and Streptomycin) against a Gram-positive bacteria (*Staphylococcus aureus*) and two Gram-negative bacteria (*Escherichia coli* and *Pseudomonas aeruginosa*), were determined (Table 1). Results illustrated that CuFe_2_O_4_@SiO_2_-Polymer showed antibacterial activity, so it’s MIC in Gram-negative and Gram-positive bacteria were 500–1000 µg/mL. Also, the results indicated that CuFe_2_O_4_ nanoparticles show more antimicrobial activity than the final composite. In fact, the release of these nanoparticles has decreased by being placed in the composite, and as a result, fewer bacteria have been destroyed.

**Table 1:** MICs in µg/mL of CuFe_2_O_4_ nanoparticles and CuFe_2_O_4_@SiO_2_-polymer against Gram-positive and Gram-negative bacteria.

Agents	MIC _mean_ $\pm$ SD (MBC _mean_ $\pm$ SD) for 3 independent tests		
	*S. aureus*	*E. coli*	*P. aeruginosa*
CuFe_2_O_4_@SiO_2_-polymer	1000 ± 0.0	510 ± 0.8	512 ± 0.34
CuFe_2_O_4_	612 ± 0.0	270 ± 0.6	253 ± 0.82
Penicillin	2.1 ± 0.0	5.9 ± 0.0	274 ± 0.7
Streptomycin	14.27 ± 0.0	3.8 ± 0.7	7.9 ± 0.52

### Evaluating synthesized nanostructure in comparison to other research

The synthesized CuFe_2_O_4_@SiO_2_-cyclic aromatic polyimide nanostructure has been found to exhibit significantly higher cell viability capability and antibacterial properties compared to previously reported studies in the literature. According to the reports regarding the cellular viability of CuFe_2_O_4_@SiO_2_-polymer nanostructure, even at a high concentration of 1000 µg/mL, cellular viability higher than 87% has been reported. Furthermore, at a lower concentration of 5 mg/mL, the cellular viability percentage of healthy HEK293T cells is close to 90%. Meanwhile, in inquiry on CuFe_2_O_4_/mesosilicalite synthesized on HFF (human foreskin fibroblasts) cell lines^46^, where nanocomposite reached only 87% viability at a concentration of only 0.1 mg/mL. In addition, it has been reported that the antibacterial properties of CuFe_2_O_4_ nanoparticles have been decreased after being covered or functionalized^47^. However, in this scrutiny, CuFe_2_O_4_@SiO_2_-polymer nanostructure has been shown to inhibit the growth of two bacterial strains, E. coli and P. aeruginosa, at a concentration of approximately 500 μg/mL, and the growth of the bacterial strain S. aureus at a concentration of approximately 1000 μg/mL. Reports have also been presented regarding composite materials containing iron nanoparticles coated with a silicate structure, with an MIC concentration of 1250 μg/ml reported for the P. aeruginosa bacterial strain^48^. Overall, the antibacterial activity and high percentage of cell viability of the nanostructure make it a promising candidate for biomedical applications, such as wound healing, infection control, drug delivery systems and tissue engineering. Therefore, the synthesized nanostructure holds great potential for various biomedical applications due to its superior properties. Further investigations are needed to explore and expand the potential applications of this novel nanostructure in biomedicine.

## Conclusions

In summary, a new nanostructure has been prepared based on CuFe_2_O_4_ MNPs and pyromellitic dianhydride. In this nanostructure, CuFe_2_O_4_ nanoparticles were used as the central core and were functionalized with TEOS, CPTMS, and phenylenediamine derivatives in separate steps. The polymerization reaction of pyromellitic dihydride was carried out on the functionalized surface of CuFe2O4 nanoparticles with phenylenediamine derivatives. Various analytical techniques such as FT-IR, TGA, EDX, FE-SEM, XRD, and VSM confirmed the synthesis of CuFe_2_O_4_@SiO_2_-cyclic aromatic polyamide nanostructure. Finally, the results of the MTT test on HEK293T healthy cells indicated that this nanostructure is biocompatible with a cell viability of 87.36% and can be suitable for in vivo use. Additionally, the antibacterial test on three bacteria strains of S. aureus, E. coli, and P. aeruginosa showed that the prepared nanostructure has antibacterial properties, and its MIC in Gram-negative and Gram-positive bacteria is between 500 and 1000 μg/mL.

## Supplementary Information

Supplementary Figures.

## Data Availability

All data generated or analysed during this study are included in this published article.
