# Supplementary material for: Fabrication and biological investigation of a novel star polymer based on magnetic cyclic aromatic polyimide chains
Source: Sci Rep. 2023 Jun 13;13:9598. doi: 10.1038/s41598-023-36619-x (PMC10264415; doi:10.1038/s41598-023-36619-x)
Supplement: Supplementary file 1 — Supplementary Figures. [file 41598_2023_36619_MOESM1_ESM.docx]

**Supporting information**

**Fabrication and biological investigation of a novel star polymer based on magnetic cyclic aromatic polyimide chains**

Reza Eivazzadeh-Keihan ^a^*, Zahra Sadat ^a^, Adibeh Mohammadi ^a^, Hooman Aghamirza Moghim Aliabadi ^b^, Amir Kashtiaray ^a^, Ali Maleki ^a^*, Mohammad Mahdavi ^c^*

*^a^Catalysts and Organic Synthesis Research Laboratory, Department of Chemistry, Iran University of Science and Technology, Tehran 16846-13114, Iran*

*^b^Advanced Chemical Studies Lab, Department of Chemistry, K. N. Toosi University of Technology, Tehran, Iran*

*^c^Endocrinology and Metabolism Research Center, Endocrinology and Metabolism Clinical Sciences Institute, Tehran University of Medical Sciences, Tehran, Iran*

**Corresponding author. E-mail*: [maleki@iust.ac.ir](mailto:maleki@iust.ac.ir)*; Fax: +98-21-73021584; Tel: +98-21-73228313.*

**Corresponding author. E-mail*: [reza.tab_chemist@yahoo.com](mailto:reza.tab_chemist@yahoo.com)

**Corresponding author. E-mail:* [momahdavi@sina.tums.ac.ir](mailto:momahdavi@sina.tums.ac.ir)

**
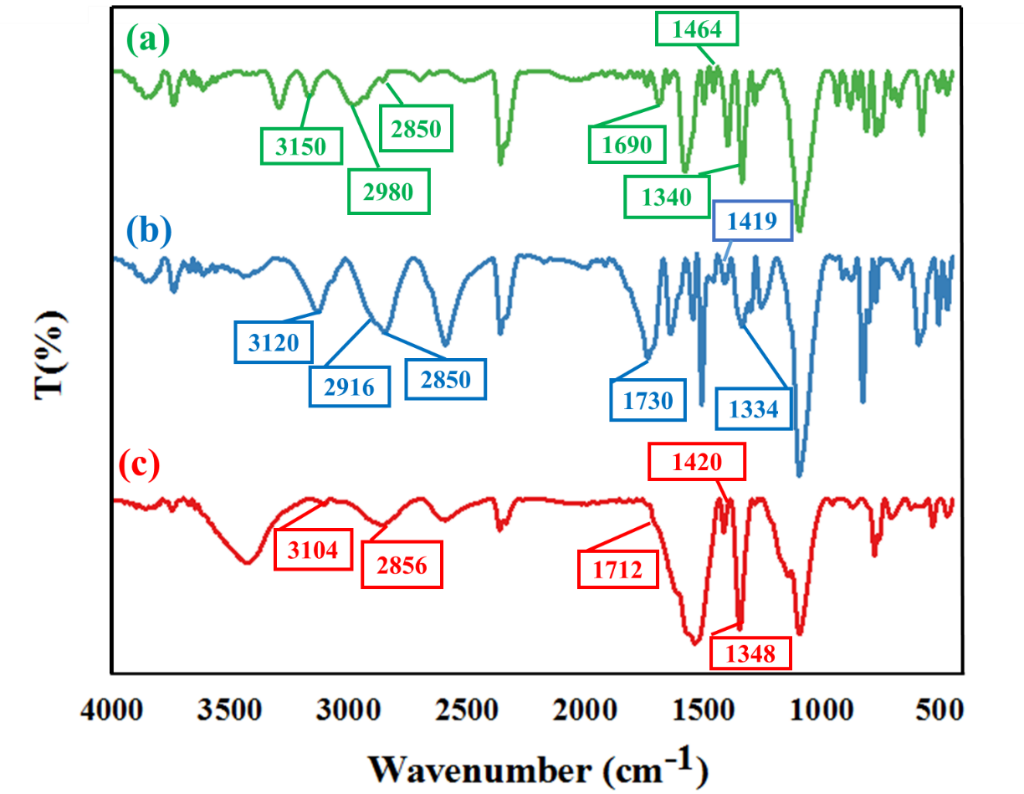
**

**Figure S1**. The FT-IR spectrums of (a) o-phenylenediamine (b) p- phenylenediamine (c) 1,5-diaminonaphthalene.


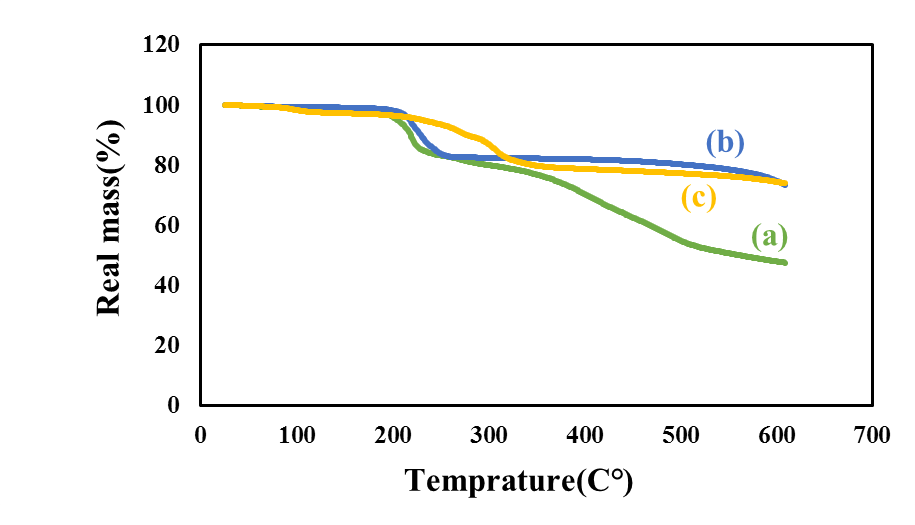


**Figure S2.** The TGA analysis of (a) o-phenylenediamine (b) p- phenylenediamine (c) 1,5-diaminonaphthalene.


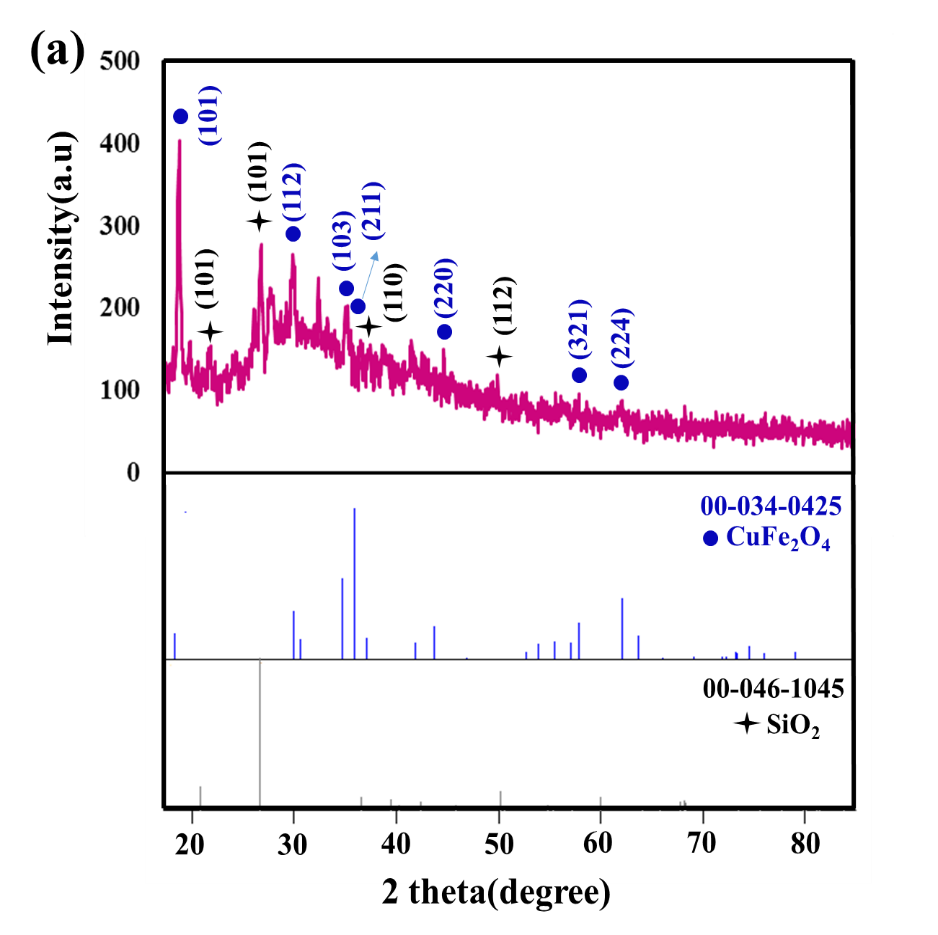

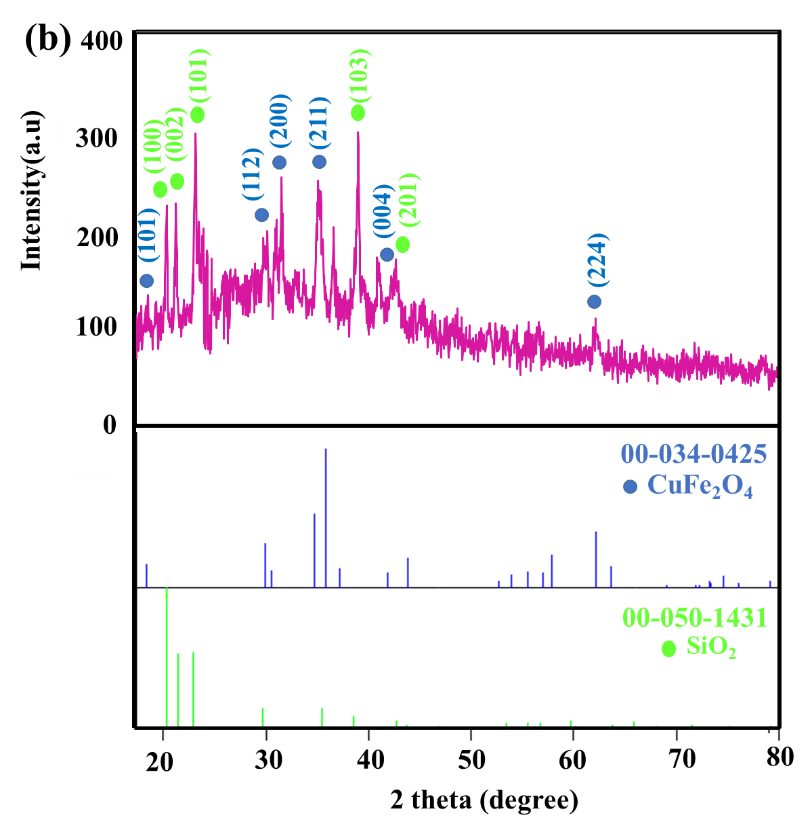

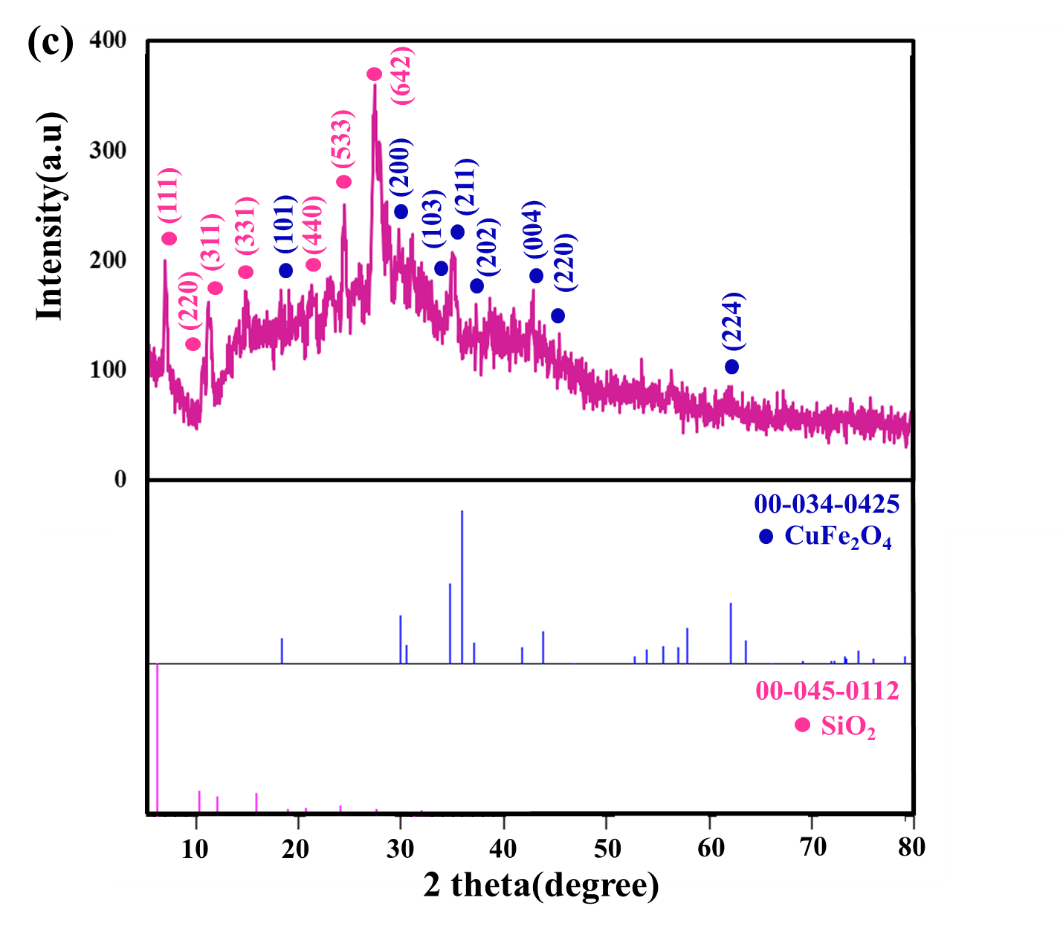


**Figure S3.** The XRD patterns of (a) o-phenylenediamine (b) p- phenylenediamine (c) 1,5-diaminonaphthalene.

**
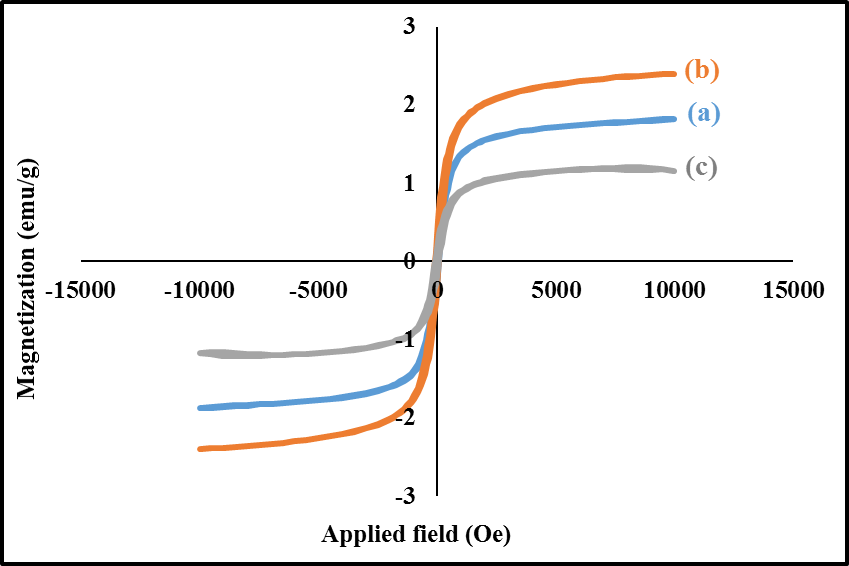
**

**Figure S4.** The VSM analysis of (a) o-phenylenediamine (b) p- phenylenediamine (c) 1,5-diaminonaphthalene.
